# A Review of the *GSTM1* Null Genotype Modifies the Association between Air Pollutant Exposure and Health Problems

**DOI:** 10.1155/2023/4961487

**Published:** 2023-02-06

**Authors:** Dwi Aris Agung Nugrahaningsih, Hevi Wihadmadyatami, Sitarina Widyarini, Rahmi Ayu Wijayaningsih

**Affiliations:** ^1^Department of Pharmacology and Therapy, Faculty of Medicine, Public Health and Nursing, Universitas Gadjah Mada, Yogyakarta, Indonesia; ^2^Department of Anatomy, Faculty of Veterinary Medicine, Universitas Gadjah Mada, Yogyakarta, Indonesia; ^3^Department of Pathology, Faculty of Veterinary Medicine, Universitas Gadjah Mada, Yogyakarta, Indonesia; ^4^Master of Biomedical Science Program, Faculty of Medicine, Public Health and Nursing, Universitas Gadjah Mada, Yogyakarta, Indonesia

## Abstract

Air pollution is one of the significant environmental risks known as the cause of premature deaths. It has deleterious effects on human health, including deteriorating respiratory, cardiovascular, nervous, and endocrine functions. Exposure to air pollution stimulates reactive oxygen species (ROS) production in the body, which can further cause oxidative stress. Antioxidant enzymes, such as glutathione S-transferase mu 1 (*GSTM1*), are essential to prevent oxidative stress development by neutralizing excess oxidants. When the antioxidant enzyme function is lacking, ROS can accumulate and, thus, cause oxidative stress. Genetic variation studies from different countries show that *GSTM1* null genotype dominates the *GSTM1* genotype in the population. However, the impact of the *GSTM1* null genotype in modifying the association between air pollution and health problem is not yet clear. This study will elaborate on *GSTM1*'s null genotype role in modifying the relationship between air pollution and health problems.

## 1. Introduction

Air pollution is a process to release pollutants into the air. Air pollutant varies significantly in the different places depending on the source of the pollution. Generally, air pollutant consists of several compounds including gaseous and particulate pollutants. Particulate pollutant or particulate matter (PM) is considered as one of the main components of a deleterious effect on health [1]. PMs could enter the tissue in the body after being inhaled, mainly due to their small size [2]. They can elicit the formation of reactive oxygen species (ROS) and, consequently, induce antioxidant enzymes expression [3]. The imbalance between ROS production and the availability of antioxidant enzymes may cause tissue damage that plays an essential role in the development of environment induced health deterioration [4].

The increase of ROS formation will stimulate antioxidant enzymes, including phase I and II metabolic enzymes [5, 6]. Glutathione S-transferases (GST), a large family of phase II enzymes, is one of the crucial enzymes modifying oxidative stress, which includes the glutathione S-transferase mu 1 (*GSTM1*), glutathione S-transferase theta 1 (GSTT1), and glutathione S-transferase pi 1 (GSTP1) classes. They provide protection toward air pollution deleterious effect. Studies have shown that genetic variation of the enzymes is associated with several air pollution diseases [7].

There are some genetic variations in GST enzymes encoding genes. One of the most common variations in the GST gene is the *GSTM1* null genotype, resulting in partial or complete loss of enzyme activity. The deletion of the *GSTM1* encoding gene is often related to poor health outcomes related to air pollutant exposure [8, 9]. In this study, we will do a review of *GSTM1* in correlation with air pollution-induced diseases.

## 2. Air Pollutant

**Figure 1:**
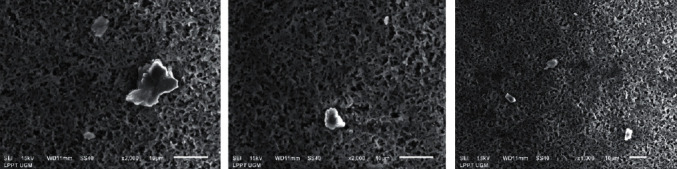
Particles from the air captured using filter nylon membrane 0.2 *μ*m and examined using SEM. Some particles have a diameter of <10 *μ*m.

Air pollutants consist of a mixture of gaseous and PM, which varies in each area depending on the source of the pollution, the wind, and sunlight. The gaseous components of air pollution mainly are carbon monoxide (CO), nitrogen oxide (NO), nitrogen dioxide (NO_2_), ground-level ozone (O_3_), and sulphur dioxide (SO_2_) [10]. PM of the air pollutant consists of solid particle and liquid droplets. Based on their size, PMs are divided into three types, such as PM10 (PM_10_), PM2.5 (PM_2.5_), and PM0.1 (PM_0.1_). PM_10_ or coarse particles are particles in the air with diameters 10 *μ*m and less, PM_2.5_ or fine particles are particles in the air with a diameter of 2.5 *μ*m and less, and PM_0.1_ or ultrafine particles are particles in the air with a diameter of 0.1 *μ*m and more diminutive [11, 12].  Figure 1 shows particles observed by scanning electron microscopy (SEM) of PM with diameter <10 *μ*m.

**Figure 2:**
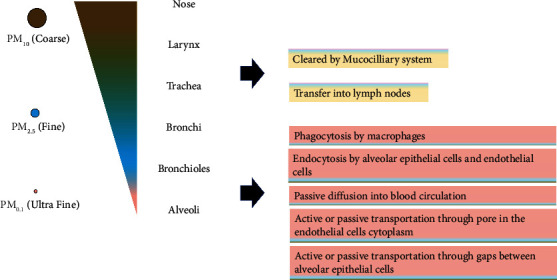
PM deposition along the airway. Coarse particles (PM_10_) and fine particles are deposited more on upper airways, cleared by the mucociliary system, and transferred into lymph nodes. Ultra-fine particles can reach alveoli, and enter blood circulation through several mechanism including phagocytosis by macrophages, endocytosis by alveolar epithelial cells and endothelial cells, passive diffusion into blood circulation, active or passive transportation through pore in cytoplasm of endothelial cells, and gaps between epithelial cells.

The size of the PM will affect their ability to penetrate the human body. Coarse materials are unable to enter the small airways and alveoli. Meanwhile, fine and ultrafine particles can enter small airways and alveoli and reach systemic circulation after penetrating the air–blood barriers [3, 13].  Figure 2 illustrates the deposition of PM_10_, PM_2.5_, and PM_0.1_ in the airway, the mechanism of clearance from the air way, and the absorption into the systemic circulation.

## 3. Air Pollutant Impact on Human Body

Based on World Health Organization estimation, exposure to air pollutants causes approximately 1 out of 10 deaths worldwide [14]. After entering the body, air pollutants, including PM, can stimulate inflammation-related agents, induce cytokines expression, trigger oxidative stress, change genes expression, and induce epigenetic changes [15]. Epigenetics changes, such as deoxyribonucleic acid (DNA) methylation may act as a mediator in the development of adverse health outcome due to PM exposure [16–18]. After entering the airway, PM increases pulmonary neutrophils, stimulates alveolar macrophage and airway epithelial cells to produce pro-inflammatory mediators, such as interleukin-6 (IL-6), interleukin-8 (IL-8), interleukin 1 beta (IL-1*β*), interleukin-4 (IL-4), tumor necrosis factor alpha (TNF-*α*), and transforming growth factor beta 1 (TGF-*β*1), which leads to local inflammation in the lung, oxidative stress, and also systemic inflammation manifesting as the increase of circulating fibrinogen, systemic IL-1*β*, IL-6, and TNF-*α* concentration [19–22].

Studies have revealed the association of PM with several diseases, from the disease of the respiratory system, which is in direct contact with PM, to the disease of the endocrine system, which is not in direct contact with PM. Respiratory system problem is the most expected system affected by PM exposure. Long- and short-term exposure to PM is related to several disease of respiratory system, such asthma, chronic obstructive pulmonary disease, and lung cancer [23]. Short- and long-term exposure to elevated PM_2.5_ levels is associated with several cardiovascular problems, such as ischemic heart disease, heart failure, and cerebrovascular disease [24–27]. Surveys conducted between 1989 and 2012 showed that PM exposure is associated with changes of atrioventricular conduction, increase of cardiovascular disease markers, and development of heart disease [20, 24, 28–31]. Exposure to PM_2.5_ is associated with Alzheimer's disease (AD), Parkinson's disease (PD), dementia, and cognitive decline [32–34]. A study in mice revealed that chronic inhalation of PM-induced oxidative stress and inflammation in the brain, increase perineuronal nets, and decrease interneurons number, which suggest PM contribution in the development of central nervous system dysfunction [35]. Furthermore, exposure to PM_2.5_ and NO_2_ in children correlates with a higher body mass index, and development of type 2 diabetes mellitus (type 2DM) by disturbing insulin sensitivity and *β*-cell function [36]. Exposure to PM_2.5_ might reduce metabolic insulin sensitivity [37]. Meta-analysis study to assess the association of PM_2.5_ exposure with type 2DM showed that long-term exposure to PM_2.5_ increases the risk of type 2 DM development [38, 39].

The length of exposure to PM affects the severity of body function disturbance. Short-term exposure to air pollution showed lower association with magnitude than long-term exposure. Long-term exposure of PM is associated with stronger systemic inflammatory marker expression than short term-exposure [22, 40]. Study in Korea showed that long-term exposure to PM_10_ was related to ischemic heart disease, but short-term exposure was not [41]. Longer duration of PM_2.5_ exposure also related to higher blood pressure [42]. *In vitro* study also showed that repeated exposure to PM_2.5_ to Beas-2B, normal bronchial epithelium cells, result in the increased sensitivity and magnitude of change of several genes associated with airway disorders [43].

## 4. Mechanism of PM Deleterious Effect to the Body

There are three hypothetical mechanisms of PM's deleterious effect on the body. The first hypothesis is that exposure of PM into the airway will elicit oxidative stress followed by lung inflammation, leading to systemic inflammation, and induce subsequent effects, such as cardiovascular inflammation, endothelial dysfunction, platelet aggregation, and vasoconstriction, which will lead to the development of multi-organ system problems. The second hypothesis is that the exposure of PM into the airway will stimulate the lung's sensory receptor, resulting in the imbalance of the autonomic nervous system, which favors sympathetic signals, leading to the increase of arrhythmia potential, an increase in heart rate and vasoconstriction. The last hypothesis suggests that the PM is translocated from the lung to the systemic circulation and directly affecting the cell by inducing systemic oxidative stress and subsequent systemic inflammation [23]. Exposure to PM_2.5_ is associated with increase inflammatory response, endothelial apoptosis, and antiangiogenic parameter expression, which could contribute to the development of atherosclerosis [44].

Oxidative stress and inflammation are two central mechanisms of PM deleterious effects to the body. *In vitro* study has revealed that alveolar macrophage isolated from humans and rats respond to PM stimulation by generating oxidants, which happens within minutes to an hour after stimulation. Meanwhile, cytokines production needs hours after PM stimulation, but the microbial origin PM can directly induces cytokines production by alveolar macrophage [45]. It is unclear which one happens first. Anyhow, oxidative stress and inflammation are related mechanisms where one phenomenon's increase will affect others [46].

## 5. PM Induces Oxidative Stress

Oxidative stress is a condition where there is an imbalance between oxidants generation and antioxidant capacity. *Oxidant* is molecules that receive electron during reductive oxidative reactions and enhances oxidation on target molecules. There are various biological oxidants, including ROS and reactive nitrogen species (RNS). Both consist of radicals and non-radicals oxidants. Radicals, molecules containing at least one electron, are more reactive and unstable compared with non-radicals oxidants. However, non-radicals oxidants also can be easily converted into radicals oxidants.  Table 1 shows radicals and non-radicals oxidants [47, 48].

Oxidants in a biological system that causes oxidative stress can be produced endogenously during biological processes and also induced exogenously due to exposure to oxidant production inducers, such as pesticide, drugs, ultraviolet light, X-ray, heavy metal, and air pollutant, including PMs [47].

Various components of PM can cause ROS and RNS generation, which leads to oxidative stress. Exposure to PM_2.5_ could cause mitochondrial damage and ROS production, which leads to cell death [49]. Exposure to PM_2.5_ in pregnant women showed positive association with placental 3-nitrotyrosine, the biomarker of nitrogen free radicals species [50]. Polycyclic aromatic hydrocarbons (PAHs), one of the PM components, generate ROS during catalytic conversion by cytochrome P450 1A1, which converts PAHs to quinones [51]. Diesel exhaust particle (DEP) components induce bioactivation by P450 reductase and generate ROS during the process. Microglia can engulf DEP entering the body and cause superoxide production. Study on alveolar epithelial cells showed that PM exposure could induce the generation of iron-derived free radicals, which can cause mitochondrial dysfunction [42, 52, 53].

**Figure 3:**
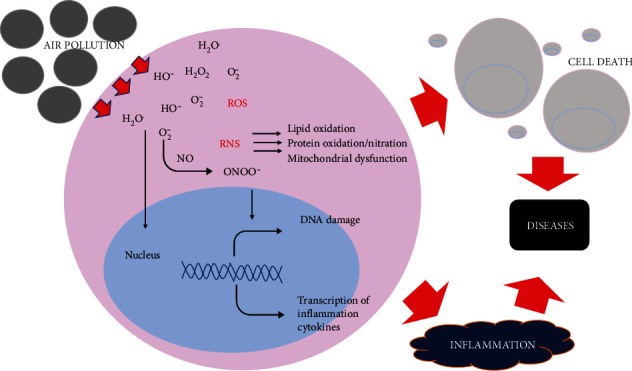
Correlation between air pollution, oxidative stress, and diseases. ROS, reactive oxygen species; RNP, reactive nitrogen species; ONOO^−^, peroxynitrite; HO^−^, hydroxyl ion; NO, nitric oxide; O_2_^−^ superoxide anion; H_2_O_2_, hydrogen peroxide.

As a response to the abundant production of ROS and RNP due to PM exposure, the body increase the production of various antioxidants, including antioxidant enzymes. This mechanism aims to maintain cellular redox equilibrium for preventing cellular injuries. However, the antioxidant defenses might not be enough when the production of ROS is chronic or the amount of exposure is enormous. When ROS production is higher and faster than antioxidant production, oxidative stress occurs [16]. Long-term exposure to oxidative stress can cause protein, lipid, and DNA, which lead to cell aging, and cell death that is important in the development of several disease, such as cancer, atherosclerosis, AD, DM, and its complications [54]. The interaction of superoxide radicals and nitrogen oxides can form peroxynitrite (ONOO^−^). The build-up of RNS can cause nitrosative stress, which is related to several diseases. Peroxynitrite (ONOO^−^) has a substantial and long-lived oxidant effect that can cause damage to DNA, membrane lipids, and protein, causing inflammation and cell death related to the initiation, progression, and severity of diseases [53–55].  Figure 3 presents the correlation between air pollution and disease development.

## 6. Endogenous Antioxidants Mechanism in Oxidative Stress

Antioxidants defense mechanisms are available throughout the body, including in bronchial lining fluid for protecting the body from the deleterious effect of PM. There are two kinds of antioxidants, synthesized and ingested antioxidants. The body could produce antioxidants in the form of protein, enzymes, and low molecular weight scavengers. Antioxidant proteins include superoxide dismutase, catalase, glutathione peroxidase, glutathione reductase, heme oxygenase-1, glutathione (GSH), and antioxidant enzymes [55]. Antioxidant enzymes include phase 1 and phase 2 metabolic enzymes. GST is one of the important phase II enzymes involved in detoxifying xenobiotics, including drugs and toxicants [56].

According to the location, GSTs in mammals consist of three families, namely cytosolic GSTs, mitochondrial GSTs, and microsomal GSTs. Microsomal GSTs are important for eicosanoids and GSH. Therefore, they are referred as membrane-associated proteins in eicosanoid and glutathione metabolism [57]. Cytosolic GSTs are more involved in the detoxification process than mitochondrial and microsomal GSTs. Cytosolic GST includes seven classes, which are classified based on their amino acid sequences. The seven classes are alpha (*α*, GSTA), mu (*μ*, GSTM), pi (*π*, GSTP), sigma (*σ*, GSTS), theta (*θ*, GSTT), omega (*ω*, GSTO), and zeta (*ξ*, GSTZ) [58]. Isoenzymes from the same class sharing more than 40% identity, and isoenzymes from different classes sharing less than 25% identity [49].

Exposure to PM could induce the expression of nuclear factor erythroid 2-related factor 2 (NRf2), important regulator of cellular resistance toward oxidants, which subsequently leading to increased expression of antioxidants, including GSTs [59]. During oxidative stress, ROS will disrupt transcription factor NRf2 binding to its negative regulator, Kelch-like enoyl-CoA hydratase-associated protein 1, in the cytoplasm leading to NRf2 translocation into the nucleus, which subsequently activates antioxidant response element and induces the transcription of several antioxidants including GSTs [60]. The expression of GSTs will help to metabolize the harmful xenobiotics into less harmful metabolites [61]. They also can conjugate GSH into a wide range of molecules, including hydrophobic and electrophilic molecules, for detoxifying them into less harmful substances and easier to be excreted out of the body [56].

## 7. Genetic Variation of Cytosolic GST

Different genes in the chromosome are responsible for different classes of cytosolic GST enzymes. They are *GSTA, GSTM, GSTP, GSTS, GSTT, GSTZ,* and *GSTO* [62]. Some of the genes encoded GST enzymes are polymorphic, resulting in the enzymes' different activity and responses to xenobiotics exposure [63]. One of the most common *GSTs* polymorphisms examined is *GSTM1* polymorphism. *GSTM1* is part of five highly similar tandem *GSTM* genes, namely *GSTM1, GSTM2, GSTM3, GSTM4*, and *GSTM5* on chromosome 1 [64]. There are several polymorphisms of GSTM. However, variation of *GSTM1*, especially deletion of *GSTM1*, is the most dominant variation among all variations of *GSTM1*.

Deletion of the *GSTM1* gene, or *GSTM1* null genotype, is highly prevalent in the population [65]. Study on Caucasian population from North America and Europe showed that the *GSTM1* null genotype frequency was 53.1%. Great Britain and Portugal had higher *GSTM1* null genotype frequency, but it was not significantly different from the rest of Caucasian population in North America and Europe. The *GSTM1* null genotype frequency in African and African American is 52.9%. Interestingly other studies showed that the frequency of *GSTM1* null genotype varies among African countries. The frequency of *GSTM1* null genotype in Egypt is 55.5%, Nigeria is 30%, Namibia is 11.2%, Cameroon is 27.8%, and Somalia is 40%. Asian population also shows difference of *GSTM1* null genotype frequency. The prevalence of *GSTM1* null genotype in Japan is 47.6%, in Korea is 52.1%, in Singapore is 56.2%, in Philippines is 51.7%, in India is 29.6%, in Afghanistan is 46%, and in Iran is 40.6% [66]. Our study conducted in Central Java, Indonesia showed that the prevalence of the *GSTM1* null genotype among Javanese is 82.1% [67]. In unrelated subjects in Iran, the prevalence of *GSTM1* null genotype is 43.8–56% [68]. Lack of *GSTM1* enzymes is known to be associated with certain diseases.

## 8. *GSTM1* Null Genotype and PM-Related Disease

*GSTM1*, as one of the essential enzymes from the GSTs family, also have an essential role in the detoxification of several toxicants from the environment, such as benzo[*α*]pyrene, nitrosamine, and aromatic amines, which also exist as PM in the air [69]. Variation of GSTs, including the *GSTM1* null genotype, have been shown to modify the body response to air pollutant exposure [70].

### 8.1. Respiratory System

Studies regarding the effect of *GSTM1* absence on air pollutant-induced respiratory system—disturbance shows contradicting results.  Table 2 shows studies about the effect of *GSTM1* null genotype on respiratory system.

The most common air pollutant studied was ozone, followed by PM. Studies with many participants, Castro-Giner et al. [87]; Curjuric et al. [79]; and Hersoug et al. [77] showed that *GSTM1* deletion did not modify the effect of air pollutant exposure on respiratory system related parameters. However, most of above mentioned studies show that *GSTM1* deletion does modify the effect of air pollutant exposure to respiratory system. Interestingly, administration of antioxidants, such as vitamins C and E above the minimum daily recommended dose might compensate for this genetic susceptibility [78]. Administration of antioxidant agents can prevent the problem of respiratory system related to air pollutant exposure.

### 8.2. Cardiovascular System

After entering the lung, PM induce cytokines and other inflammatory factors, which consequently cause local lung inflammation and further cause systemic inflammation. This condition is often related to the increased of cardiovascular disease [88–90]. Several studies revealed the effect of *GSTM1* null genotype in modifying air pollutants, especially PM, association with cardiovascular parameters.  Table 3 showed a summary of studies that investigate *GSTM1* null genotype effect on PM-related cardiovascular parameters.

Among nine studies presented in Table 3, six studies showed that *GSTM1* modify the interaction between air pollutant with cardiovascular marker namely heart rate variability, brachial artery dilatation, and soluble vascular cell adhesion molecule (sVCAM-1). Meanwhile, another three studies showed that *GSTM1* deletion has no effect on air pollutant correlation with several cardiovascular parameter, blood markers of systemic inflammation, prothrombotic state, oxidative stress, vascular dysfunction, and blood pressure. The increase of cardiovascular disease-related marker associated with air pollutant exposure in individuals with *GSTM1* null genotype suggest that individuals with deletion of *GSTM1* is more prone to develop air pollutant induce cardiovascular. Interestingly, the administration of statin could modulate the *GSTM1* deletion effect associated with cardiovascular disturbance and air pollutants [70]. Those results show a potential intervention to prevent cardiovascular disturbance associated with air pollutants in individuals with *GSTM1* null genotype.

### 8.3. Endocrine System

There are only a few studies about the *GSTM1* null genotype effect on air pollution-induced endocrine system disease.  Table 4 showed a summary of studies about *GSTM1* null genotype effect on air pollution-induced endocrine system disturbances.

Based on those results, *GSTM1* null genotype is an essential factor in the glucose metabolism disturbance and inflammation development in air pollutant-induced DM. Individual carrying *GSTM1* null genotype is more prone to develop glucose metabolism disturbance, and when they are DM patients, they might be easier to experience DM-related complications elicited by exposure to air pollutants.

### 8.4. Nervous System

*GSTM1* null genotype also modifies the association of air pollutants with several nervous system problems.  Table 5 summarizes studies about *GSTM1* null genotype effect on air pollutant-induced nervous system diseases.

That studies showed that *GSTM1* variation could modify air pollutant effect on nervous system disturbance.

## 9. *GSTM1* Null Genotype and Antioxidant

DEP exposure to primary human bronchial epithelial cell with *GSTM1* deletion result in markedly higher IL-8 and IL-1*β* expression compared with than those on primary human bronchial epithelial cell without *GSTM1* deletion. Interestingly, antioxidants like *N*-acetyl cysteine can inhibit the DEP-induce cytokines expression in primary human bronchial epithelial cells [103]. This result showed that some substances could modify the effect *of GSTM1* deletion.

Antioxidants are essential factors for protecting the body from the deleterious effects of oxidants. The antioxidant can be produced endogenously by endogenous enzymes, such as GSTs enzymes or exogenously by consuming antioxidants from diet. Individuals with endogenous antioxidant deficiencies, such as in *GSTM1* null genotype person will benefit from higher dietary antioxidant consumption. Consumption of antioxidant-rich diet in *GSTM1* null genotype individual has been known to prevent or reduces DNA adduct formation [104]. Antioxidant supplementation has a stronger effect on modifying the deleterious effect of ozone on asthmatic children with *GSTM1* null genotype than on asthmatic children with intact *GSTM1* genotype [78]. In hypertensive patients, the administration of kale juice results in lower DNA damage, especially in individuals with *GSTM1* null genotype [49].

Studies on smokers showed that people with *GSTM1* null genotype develop faster atherosclerosis progression than people with *GSTM1* wild type. Vitamin E supplementation seems to reduce the atherosclerosis progression better in *GSTM1* null genotype individuals [105]. Study on relation between *GSTM1* and lung cancer on smokers showed that the risk of lung cancer is higher among smokers with *GSTM1* null genotype and not receiving *α*-tocopherol supplementation compare with those on smokers with *GSTM1* null genotype and receiving *α*-tocopherol supplementation. However, *β*-carotene supplementation does not modify the correlation between *GSTM1* and the risk of lung cancer development [106]. Clinical study of vitamin B supplementation on healthy adults exposed to PM_2.5_ for 2 hours showed that vitamin B supplementation could mitigate PM_2.5_ effects on heart rate, LF power, total white blood count, and lymphocyte count [107]. Those studies suggest that antioxidant supplementation is potential way to modify the PM exposure effect on the development of system organ dysfunction in individual with *GSTM1* null genotype.

## 10. Conclusion

*GSTM1* deletion modify the effect of air pollutants, including PM, on the development of several health problems through oxidative stress modulation. Several substances, such as antioxidants, can potentially attenuate the deleterious effect of air pollutants on the body in individuals with *GSTM1* null genotype. However, further study is needed to explore the benefit of antioxidants supplementation to prevent development of system organ problems related to PM exposure.

## Figures and Tables

**Table 1:** Example of ROS and RNS radicals and non-radicals oxidants.

Reactive oxygen species (ROS)	Radicals oxidants: superoxide (O_2_^−^), hydroxyl radical (OH^−^), and hydroxyperoxyl (H_2_O^−^)	Non-radicals oxidants: hydrogen peroxide (H_2_O_2_), ozone (O_3_), and organic peroxide (ROOH)
Reactive nitrogen species (RNS)	Radicals oxidants: nitric oxide (NO^−^) and nitrogen dioxide (NO_2_^−^)	Non-radical oxidants: nitrous acid (HNO_2_), peroxynitrite (ONOO^−^), and dinitrogen trioxide (N_2_O_3_)

**Table 2:** Studies of the effect of *GSTM 1* absence on respiratory system.

No.	Author	Sample number	Pollutant type	Parameters measured	Result
1.	Yang et al. [30]	1180	PM_2.5_	Low respiratory tract infection (LRTI)	*In utero* exposure to PM_2.5_, especially during the third trimester of pregnancy, is associated with a higher frequency of lower respiratory tract problems at one year, especially in the absence of *GSTM1* in the mother
2.	Reddy et al. [71]	129	PM_10_, SO_2_, NO_2_, and NO	Percent change in intraday variability of forced expiratory volume 1 (FEV)	Higher exposure to SO_2_ associates with more significant FEV1 intraday variability on those with *GSTM1* deletion
					Children with *GSTM1* positive genotype associate with
3.	Romieu et al. [72]	151	Ozone	Respiratory symptoms and lung function	Asthmatic children with *GSTM1* null genotype showed increase of reported breathing difficulty associated with ozone exposure
4.	Alexis et al. [73]	35	Ozone	Lung function and inflammation	*GSTM1* did not modify lung function and granulocyte influx after acute ozone exposure, but *GSTM1* null genotype person showed a significant increase of airways neutrophils and increase expression of HLA-DR 24 hours after ozone exposure
5.	Framptom et al. [74]	24	Ozone	Pulmonary, systemic vascular function, and cardiac function	There were no consistent effects of ozone exposure with all parameters measured. All the results were not dependent on the *GSTM1* genotype
6.	Yang et al. [30]	307	Indoor PM_2.5_ and environmental tobacco smoke (ETS)	Susceptibility to RTIs	Deletion of *GSTM1* increases the susceptibility of RTIs associated with prenatal exposure of indoor PM_2.5_ and ETS
7.	Kim et al. [75]	59	Ozone	Pulmonary function and subjective symptom	*GSTM1* genotype alone did not modify ozone induces an increase in neutrophilic inflammation in the airways and decrease of FEV1
8.	Ghosh et al. [76]	793	Second-hand smoke (SHS), polycyclic aromatic hydrocarbons (PAH), and PM_2.5_	Acute bronchitis	*GSTM1* genotype did not modify the risk to develop acute bronchitis in preschool children after exposure to SHS, PAH, and PM_2.5_
9.	Hersoug et al. [77]	3471	The indoor source of PM	Objective markers of respiratory disease	*GSTM1* genotype did not modify the change of objective markers of respiratory disease after exposure to the indoor source of PM
10.	Romieu et al. [78]	158	Ozone	Forced expiratory flow	Asthmatic children with *GSTM1* null genotype were more prone to deleterious effects on airways related to ozone exposure
11.	Curjuric et al. [79]	4365	PM_10_	Lung function	*GSTM1* genotype did not modify the change of lung function after exposure to PM_10_
12.	Madden et al. [80]	15	Diesel exhaust (DE) and ozone	Lung function	*GSTM1* genotype did not modify the changes in lung function associated with DE and ozone exposure
13.	Lan et al. [81]	244	Indoor smoky coal emission	Lung cancer	*GSTM1* null genotype is associated with a higher risk of lung cancer in smoky coal use.
14.	Dey et al. [82]	155	They were non-respirable PM, SO_2_, NO_2_, organic silicone, and aliphatic C–F compounds in the air.	Lung function	*GSTM1* null genotype modify the changes of lung function in smokers living around coal mines.
15.	Bowatte et al. [83]	620	Traffic-related air pollution (TRAP)	Asthma, wheeze, and hay fever	*GSTM1* null genotype modifies the risk of asthma and wheeze, but not hay fever related to TRAP exposure during the first year of life
16.	Chen et al. [84]	210	Ozone	Lung function	*GSTM1* null/*NQO1* Pro187 Pro-combination, but not *GSTM1* null genotype alone is associated with ozone-related changes in lung function
17.	Bergamaschi et al. [85]	24	Ozone	Lung function and blood parameter	Participants with *NOQ1* WT and *GSTM1* null show lung function and serum CC16 change associated with ozone level
18.	Dillon et al. [86]	35	Clinical Center Reference Endotoxin (CCRE)	Airway and systemic inflammation parameter	The participant with *GSTM1* null genotype shows a significant increase of circulating white blood cells, polymorphonuclear neutrophils, platelets, and sputum after the challenge with CCRE
19.	Castro-Giner et al. [87]	2920	Local traffic-related air pollution (estimated NO_2_)	Asthma	*GSTM1* null genotype did not modify the effect of local traffic-related air pollution exposure with asthma prevalence
20.	Zhang et al. [31]	17	Diesel exhaust (DE) and allergen	Lung function	*GSTM1* genotype did not modify the change of lung function after exposure to DE or allergen

**Table 3:** Studies about *GSTM1* null genotype effect on air pollutant-induced cardiovascular changes.

No.	Author	Sample number	Pollutant type	Parameters measured	Result
1.	Chahine et al. [91]	539	PM_2.5_	Heart rate variability (HRV): standard deviation normal-to-normal (SDNN), high frequency (HF), and low frequency (LF)	*GSTM1* modifies the association of PM_2.5_-HRV. In *GSTM1* null genotype participants, PM_2.5_ concentrations negatively modify SDNN, HF, and LF
2	Meier-Girard et al. [92]	1593: Total, 510 (without cardiovascular morbidity)	TPM_10_	HRV/HRD	There is a strong association between TPM10 and HRV/HRD parameters in participants with *GSTM1* null genotype
3	Ren et al. [93]	1000	PM_2.5_ and black carbon (BC)	Homocysteine	*GSTM1* null genotype does not modify adjusted percent change in homocysteine (log) association with interquartile range increases of 7-day moving averages of PM_2.5_ and BC
4	Probst-Hensch et al.[94]	1133	Second-hand smoke (SHS)	SDNN and standard deviation of all normal-to-normal intervals (SDAN)	There is a suggestion for an elevated decrease in SDNN and SDAN among subjects exhibiting *GSTM1* deletion genotype and high SHS exposure or obesity
5	Schwartz et al. [70]	2280	PM2.5	HF component of HRV	There is a significant association of PM_2.5_ and HF in participants with the *GSTM1* null deletion
6	Sack et al. [95]	21	Diesel exhaust particle	Brachial artery diameter (bad)	Participants with *GSTM1 null genotype* showed more significant vasoconstriction of bad after DE exposure than subjects with the wild-type allele
7	Madrigano et al. [29]	809	BC	Soluble intercellular adhesion molecule (sICAM-1) and soluble vascular cell adhesion molecule (sVCAM-1)	They found evidence for differential effects of BC exposure on the change of % sVCAM-1 by *GSTM1* status
8	Balmes et al. [96]	87	Ozone	Blood markers of systemic inflammation, prothrombotic state, oxidative stress, and vascular dysfunction	*GSTM1* did not modify the interaction between ozone and systemic inflammation, oxidative stress, and endothelial dysfunction marker
9	Mordukhovich et al. [97]	457	BC	Systolic Blood Pressure (SBP) and Diastolic Blood Pressure (DBP)	*GSTM1* null genotype did not modify the interaction between BC and blood pressure

**Table 4:** Studies about GSTM1 *null genotype* effect on air pollutant especially PM association with diabetes mellitus (DM).

No.	Author	Sample number	Pollutant type	Parameters measured	Result
1	Kim et al. [98]	560	PM_10_, ozone, and NO_2_	Fasting glucose, insulin, homeostatic model assessment (HOMA) index to assess insulin resistance (IR)	PM_10_, ozone, and NO_2_ induced fasting plasma glucose, insulin, and HOMA associated interquartile range increase in participants with *GSTM1* null genotype compared with those with intact *GSTM1*
2	Schneider et al. [99]	20 Type 2 diabetic mellitus	PM_2.5_	Monocyte marker CD40 and CD80	Markers CD40 and CD80 show a stronger association with PM_2.5_ in individuals with *GSTM1* null genotype
3	Schneider et al. [100]	22 Type 2 diabetic mellitus	PM_2.5_	Inflammation/coagulation parameters and blood panel	In individuals with DM with *GSTM1* null genotype, the changes in IL-6, RBC, total haemoglobin, and Mean corpuscular volume (MCV) induced by PM_2.5_ is more prominent

**Table 5:** Studies about *GSTM1* null genotype effect on air pollutant-induced nervous system disturbances.

No.	Author	Sample number	Pollutant type	Parameters measured	Result
1.	Lee et al. [101]	720 pregnant women	Second-hand smoke (SHS)	Mental Development Index (MDI) and Psychomotor Development Index (PDI) in their children	MDI of children from mothers with *GSTM1*/GSTT1 double deletion showed a significant negative association with cotinine level (predictor for SHS exposure)
2.	de Souza Pinhel et al. [102]	423	Pesticide exposure	Parkinson's disease (PD)	A combination of *GSTM1*–*GSTT1* null genotype modifies PD risk related to pesticides exposure
